# Vitreous Transcriptomics in Diabetic Retinopathy: Current Research and Future Prospects

**DOI:** 10.3390/biology15211997

**Published:** 2026-11-04

**Authors:** Emilia A. Bober, Maya Natasha Thomas, Małgorzata Woronkowicz, Conor M. Ramsden

**Affiliations:** 1 Torbay Hospital, Torbay and South Devon National Health Service Foundation Trust, Torquay TQ2 7AA, UK; 2 Musgrove Park Hospital, Somerset National Health Service Foundation Trust, Taunton TA1 5DA, UK; mayanatasha.thomas@nhs.net; 3 North Devon District Hospital, Royal Devon University Healthcare National Health Service Foundation Trust, Barnstaple EX31 4JB, UK; malgorzata.woronkowicz.14@alumni.ucl.ac.uk; 4 West of England Eye Unit, Royal Devon University Healthcare National Health Service Foundation Trust, Exeter EX2 5DW, UK; 5 Faculty of Health and Life Science, University of Exeter Medical School, Exeter EX1 2HZ, UK

**Keywords:** diabetic retinopathy, vitreous humour, transcriptomics, microarrays, next-generation sequencing

## Abstract

Diabetic retinopathy (DR) is a major cause of preventable blindness in working-age adults, yet the molecular mechanisms behind its vision-threatening complications remain not fully understood. Transcriptomics enables the study of these processes through active gene expression, providing a pathway to the discovery of new biomarkers and therapeutic targets. Vitreous humour is commonly affected in numerous retinal pathologies, including DR, and is accessible through pars plana vitrectomy, making it a good candidate for transcriptomics research. Following PubMed and Embase search, 18 articles on vitreous transcriptomics were included. They were subclassified into themes of non-coding RNA, RNA methylation, angiogenesis, inflammation and fibrosis. We concluded that vitreous transcriptomics has the potential to influence future DR diagnostics and therapeutics. However, current evidence is limited by underpowered studies and lack of standardised protocols.

## Simple Summary

Diabetes can damage the blood vessels at the back of the eye, resulting in a condition called diabetic retinopathy. Globally, it is a leading cause of sight loss among working-age adults. The eye is filled with a transparent gel known as the vitreous, which can be affected in diabetic retinopathy. It is now possible to determine the genetic composition of the vitreous, revealing which genes are active or inactive during disease. We examined 18 articles that employed this approach in individuals with diabetic retinopathy. Together, they identified genes associated with new blood vessel formation, scarring, and inflammation, some of which might serve as targets for new therapies or diagnostics. However, the studies were small and employed different methods, making their results hard to compare. Larger, standardised studies are necessary before this approach can be useful for patient care.

## 1. Introduction

Diabetes mellitus (DM) affects approximately 537 million people worldwide [1]. It is a systemic disease that affects many vital organ systems, including the eyes, leading to considerable morbidity. Diabetic retinopathy (DR) is one of the earliest microvascular complications of DM. It affects 1 in 3 individuals with the disease and is one of the leading causes of preventable vision loss in the working adult population [2].

Clinically, DR is classified into non-proliferative diabetic retinopathy (NPDR) and proliferative diabetic retinopathy (PDR). The main cause of visual deterioration in DR is diabetic macular oedema characterised by sub- or intraretinal fluid accumulation at the macula. Other sight-threatening sequelae of DR directly driven by neovascularisation and retinal ischaemia include vitreous haemorrhage, tractional retinal detachment and neovascular glaucoma. Understanding the pathophysiological processes behind DR and its sequelae is crucial for future advancements in targeted treatment options.

In recent years, transcriptomics has become a means of studying disease processes at the genetic level. The transcriptome, which is a collection of RNA molecules present in an organism at a specific point in time, represents the subset of genes that are actively expressed in a particular cell or tissue type. Its composition is dynamic and can be influenced by developmental stage, environmental factors and physiological conditions [3].

Recent studies have generated atlases of gene expression, focusing on the posterior and anterior segments of the eye [4,5,6,7,8]. However, human vitreous transcriptomics remains an understudied area that requires further attention. Vitreous humour occupies four-fifths of the human eye and provides both structural and biochemical support to the adjacent ocular structures [9]. From previous studies, it is understood that the composition of vitreous can change in various retinal disease states, including DR, due to its proximity and interplay with inner retinal structures. Sampling of vitreous is possible as it is a surgically accessible reservoir. Identifying the transcriptome of diseased vitreous can therefore provide an opportunity for advancing the diagnostics and therapeutics of the vision-threatening sequelae of DR.

In this narrative review, we summarise some of the key findings in current vitreous transcriptomic research.

## 2. Background

### 2.1. Brief Overview of the Pathophysiology of Diabetic Retinopathy

Hyperglycaemia is the primary biochemical derangement driving the pathophysiology of diabetes. In the early stages of DR, high blood glucose concentration triggers oxidative stress induced by accumulation of advanced glycation end products (AGEs), activation of protein kinase C pathways, and disruption of the polyol pathway and hexosamine pathway [10]. This biochemical dysregulation contributes to apoptosis of specialised perivascular mural cells called pericytes [11]. In health, pericytes are responsible for maintaining the integrity of capillary walls. Loss of capillary wall strength leads to focal vascular weakness and the formation of outpouchings, called microaneurysms [12], which are hallmark findings in early stages of NPDR.

**Figure 1:**
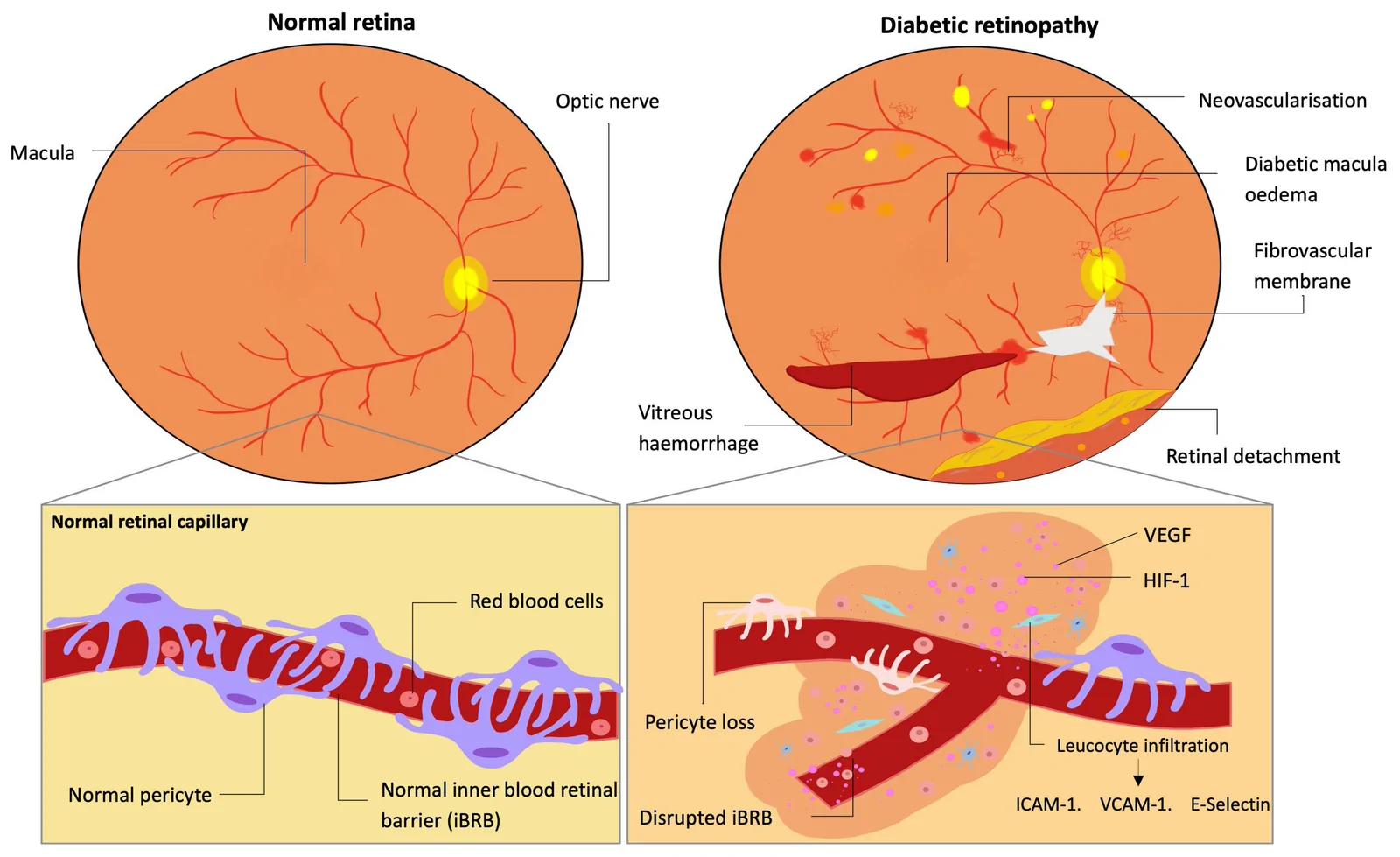
Normal retina and retinal vasculature on the left and complications of diabetic retinopathy on the right.

Sustained hyperglycaemia further drives apoptosis of endothelial cells and thickening of the basement membrane. Those structural changes disrupt the inner blood–retinal barrier (iBRB) and eventually lead to capillary occlusion and ischaemia [13]. Lack of oxygen triggers upregulation of angiogenic factors, such as vascular endothelial growth factor (VEGF) and hypoxia-inducible factor 1, resulting in the formation of new blood vessels (neovascularisation), which is a characteristic finding in proliferative disease [10]. These new blood vessels are fragile and often bleed into the vitreous, leading to sight-threatening sequelae such as severe vitreous haemorrhage and secondary glaucoma (Figure 1).

Moreover, in the absence of complete posterior vitreous detachment, the new blood vessels continue to grow into the posterior vitreous, using it as a scaffold and form fibrovascular membranes (FVMs). This fibrous proliferation and vitreous contraction may cause traction on the retina, pulling the neurosensory retina from the retinal pigment epithelium and causing a retinal detachment, potentially leading to significant visual impairment if the macula is involved.

Chronic low-grade inflammation triggering leukostasis is another important feature of DR, leading to capillary blockage and cell death [14]. The mechanism of inflammation in DR is complex and believed to be driven by AGEs, oxidative stress and upregulation of adhesion molecules in endothelial cells. Previous immunoassay studies have shown that molecules such as intercellular adhesion molecule-1 (ICAM-1), vascular cell adhesion molecule-1 (VCAM-1), and selectins (E-selectin) are overexpressed in PDR and facilitate leukocyte–endothelial adhesion [15]. Chemokines such as monocyte chemotactic protein-1, macrophage inflammatory protein-1α (MIP-1α), and MIP-1β have also been shown to contribute by promoting leukocyte recruitment and activation to areas of hypoxic retina [16]. Moreover, glial cells in the retina, including astrocytes, Müller cells and microglia, release proinflammatory molecules under hyperglycaemic stress, further exacerbating the neuroinflammatory process [17].

### 2.2. Transcriptomics

**Figure 2:**
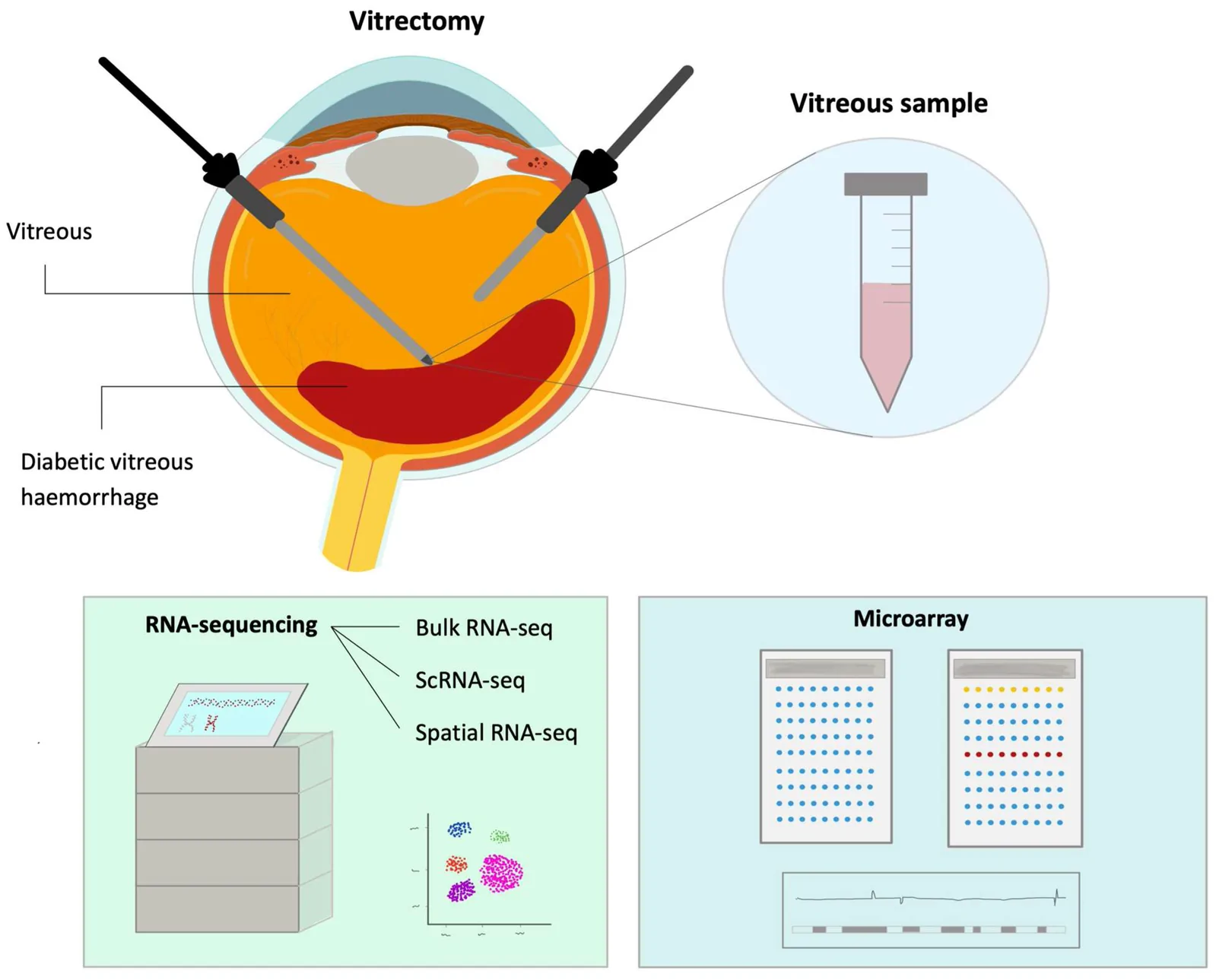
Simplified schematic depicting vitreous biopsy for microarray or RNA-sequencing methods.

Transcriptomics is a study of all RNA transcripts expressed from an organism’s genome. The field of transcriptomics has seen major advancements in recent decades. Its emergence can be traced back to the 1970s when low-throughput Sanger sequencing was first introduced [18]. However, this approach only captured isolated DNA fragments, which made whole-genome sequencing slow and labour-intensive. Two decades later, the microarray method was introduced, allowing for the simultaneous sequencing of thousands of transcripts. This was followed by the advent of next-generation sequencing techniques, such as RNA sequencing (RNA-seq) in the 2000s, which has revolutionised the field, enabling efficient quantification of entire human transcriptomes in a matter of hours rather than years [18] (Figure 2).

Furthermore, single-cell RNA sequencing (scRNA-seq) is now possible, superseding microarray and bulk RNA-seq. It identifies each cell’s transcriptional pattern and uncovers the heterogeneity in gene expression and regulation within a particular cell group. Importantly, it is a tool for identifying rare cell populations, biomarkers, and transcriptional responses to pharmacological agents. Although it is still a recently developed technique, scRNA-seq has already been successfully applied in a clinical setting, guiding therapeutic intervention in a patient with refractory drug-induced hypersensitivity syndrome [19]. In addition, microarray assays are being trialled in the field of oncology for prognostication [20,21] and diagnostics [22,23] of certain types of cancers.

Several technical challenges related to current transcriptomic methods have been well-described in the literature [24,25,26,27]. Some of the important considerations are outlined in Table 1. Although RNA-seq dominates the current era of transcriptomics, microarrays remain a valuable method. Arrays are more standardised and cost-effective, which makes them good candidates for use in clinical practice. The duration of array processing, from sample collection to data generation, varies and can range from hours to several days, depending on the circumstances [28]. If sample processing and testing procedures are standardised, there is potential to reduce the testing cycle to 24 h in the future, supporting rapid clinical decision-making. Artificial intelligence is also being trialled as a tool to facilitate analysis of big datasets generated during the sequencing process [29]. Combined, these tools have potential to improve patient outcomes by allowing for a more tailored approach to diagnostics and treatment.

## 3. Methods

**Figure 3:**
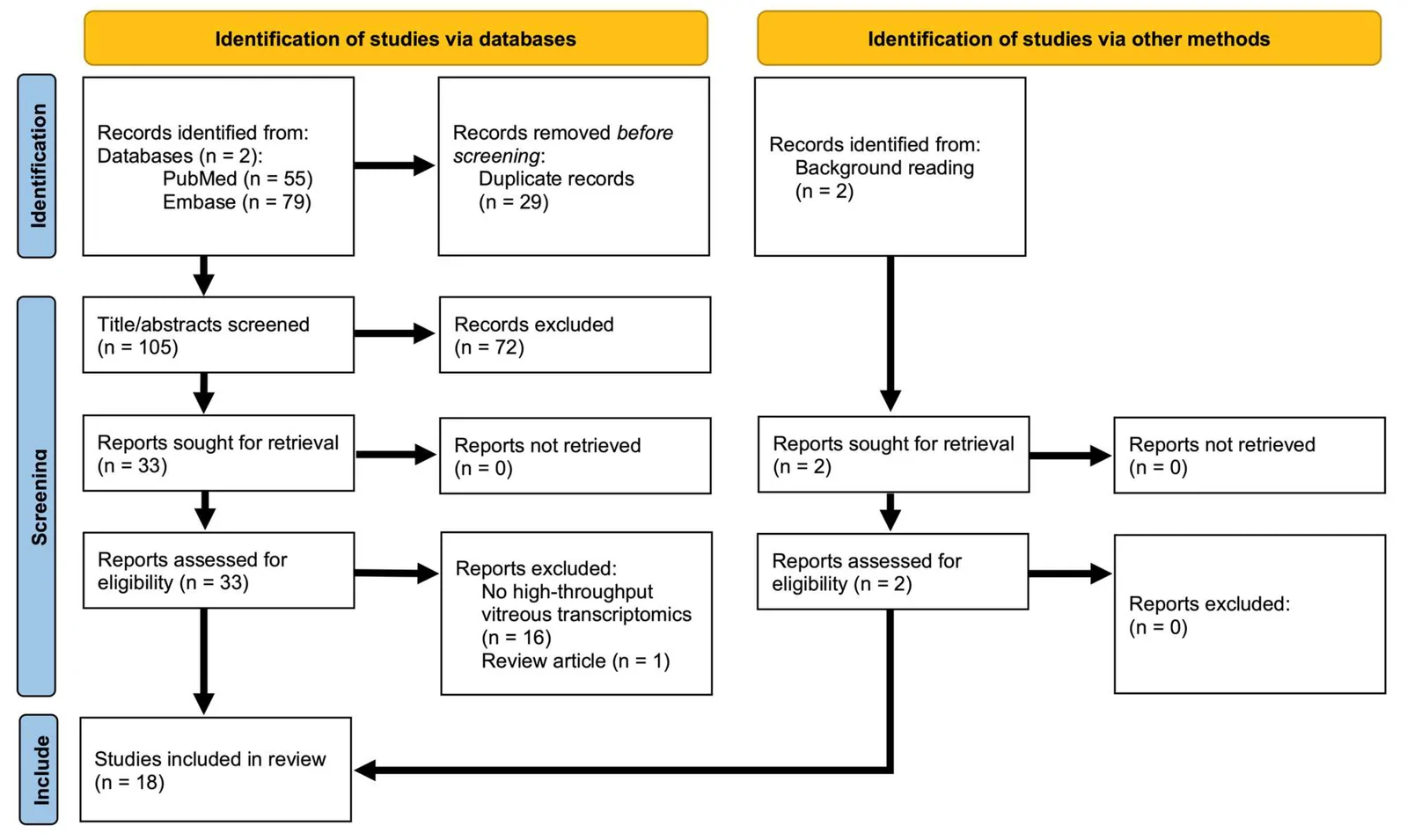
PRISMA 2020 flow diagram demonstrating the literature search process [31].

In this narrative review, two electronic databases were searched: PubMed and Embase (via Ovid). The search was carried out across three concept blocks: diabetic retinopathy, vitreous, and transcriptomic methodology. Records were excluded if they were reviews, conference abstracts, duplicates or if the study did not involve vitreous transcriptomics or did not investigate diabetic retinopathy. Studies reporting the transcriptomics of FVMs were retained, as these structures reside within the vitreous cavity. Only abstracts written in English were considered. Screening of title/abstracts was performed by a single reviewer. A full-text review of the identified abstracts meeting the inclusion criteria was subsequently conducted by the same reviewer. The summary of the article screening process is illustrated in Figure 3.

The search string was created by generative artificial intelligence (genAI) chat (Claude Opus 5, Anthropic, San Francisco, CA, USA, accessed 27 August 2026). It was then reviewed and manually edited by the authors prior to execution. The original prompt and output can be found in Supplementary File S1. The final search string for PubMed was as follows:

(“Diabetic Retinopathy”[MeSH] OR diabetic retinopathy[tiab] OR proliferative diabetic retinopathy[tiab]) AND (“Vitreous Body”[MeSH] OR Vitrectomy[MeSH] OR vitreous[tiab] OR vitreous humor[tiab] OR vitreous fluid[tiab] OR vitrectomy[tiab]) AND (Transcriptome[MeSH] OR “Gene Expression Profiling”[MeSH] OR “Sequence Analysis, RNA”[MeSH] OR transcriptomic*[tiab] OR transcriptome[tiab] OR “RNA-seq”[tiab] OR “RNA sequencing”[tiab] OR “gene expression profiling”[tiab] OR microarray[tiab] OR “differentially expressed genes”[tiab])

The search retrieved 55 records, out of which 15 were included, spanning publication years 2015–2026.

A supplementary search of Embase (Ovid) was subsequently carried out using the following search string, which was initially created by genAI (Claude Opus 5, Anthropic, accessed 27 August 2026) and subsequently manually edited by the authors prior to execution (Supplementary File S1):

1. exp diabetic retinopathy/
2. diabetic retinopath*.ti,ab,kw.
3. exp vitreous body/
4. (vitreous adj3 (humo*r or fluid or body)).ti,ab,kw.
5. vitrectomy.ti,ab,kw.
6. transcriptomics/or transcriptome/or gene expression profiling/or RNA sequencing/or RNA sequence/or microarray/
7. (transcriptomic* or transcriptome* or “RNA seq*” or RNAseq or “gene expression profil*” or microarray or “differentially expressed gene*”).ti,ab,kw.
8. or/1–2 [**DR]
9. or/3–5 [**vitreous]
10. or/6–7 [**transcriptomics]
11. 8 and 9 and 10

The search retrieved 79 records. Screening was performed by a single reviewer against the criteria described above, and one additional article was included.

Two further articles were identified outside the database searches. Those were recommended by a co-author through background reading. They were assessed against the same eligibility criteria, giving a final total of 18 included studies.

## 4. Vitreous Transcriptomics and Key Thematic Findings

The summary of included studies can be found in Table 2.

### 4.1. Angiogenesis and Neovascular Signalling

In PDR, the primary goal of the current treatment is to suppress the angiogenic drive. Therefore, identifying treatment targets beyond VEGF is important in expanding future therapeutic options. In recent years, A disintegrin-like metalloprotease with a thrombospondin type 1 motif, 13 (ADAMTS13), has been shown to regulate inflammation, angiogenesis, and ECM degradation [50]. Li et al. carried out RNA-seq on human vitreous samples. They studied 5 PDR cases and 5 controls (please refer to Table 2 for sample sizes and types for the included studies). They detected upregulation of ADAMTS13 in PDR [32]. They also demonstrated that ADAMTS13 expression promoted angiogenesis by upregulating the polo-like kinase-1/angiopoietin-2 signalling pathway, which was independent of VEGF. These findings suggested that targeting ADAMTS13 may provide an alternative solution for patients with suboptimal response to anti-VEGF therapy.

### 4.2. Inflammation

The immunological landscape of the PDR vitreous was investigated by Haliyur et al., who conducted the first human vitreous transcriptomic characterisation at the single-cell level [33]. Vitreous samples were obtained from dilute cassette washings of patients with advanced haemorrhagic PDR, and droplet-based scRNA-seq was subsequently carried out. Peripheral blood samples collected from the same subjects acted as controls. Results revealed a high abundance of T cells and an almost complete absence of neutrophils in the vitreous compared to peripheral blood. These findings generated interest since there is currently no definitive agreement on the exact role of adaptive immunity in PDR. Yet, its presence in diseased vitreous could be of clinical relevance and facilitate the development of future immunomodulatory treatments.

Boneva et al. carried out RNA-seq analysis, primarily focusing on the role of hyalocytes in vitreous PDR [34]. Hyalocytes are specialised resident macrophages that live in the vitreous close to the vitreo–retinal interface. In hyperglycaemia, they are believed to drive angiogenesis, release profibrogenic factors and contribute to erythrophagocytosis [51]. The results revealed the presence of increased proinflammatory and proangiogenic factors, such as interleukin-6 and angiopoietin-2, in PDR hyalocytes compared to the hyalocytes of patients with epiretinal membrane (ERM) and macular hole (MH). Therefore, immunomodulation of hyalocytes could be a potential future therapeutic option for treating PDR.

The immunological landscape of the PDR vitreous was also investigated by Hu et al. [35]. Transcriptional analysis through snRNA-seq revealed microglia to be the major cell subset of the FVMs. A secondary analysis of their dataset (GSE165784) by Scheri et al. challenged the initial findings [52]. They could not reproduce the microglial subpopulation Hu et al. described, and they were unable to replicate the profibrogenic properties in any cell cluster. Instead, they found that FVM samples contained macrophages rather than microglia. In response, the original authors acknowledged that their labelling could not exclude monocyte-derived macrophages [53]. They argued that transcriptomics cannot resolve this dispute, as microglia are capable of exhibiting heterogeneous disease-associated profiles in response to local insults. Therefore, a future lineage tracing experiment should be planned to determine the origin of FVM-resident myeloid cells.

### 4.3. Fibrosis and ECM Remodelling

The presence of FVMs is a characteristic feature of severe PDR. The FVMs consist of stromal tissues and cellular components, such as retinal glial cells, macrophages/hyalocytes, vascular endothelial cells and fibroblasts [54]. The FVMs can contract and induce tractional retinal detachment leading to vision loss. Despite their high importance in the disease process, the mechanisms underlying FVM development and progression are not fully understood. Ishikawa et al. [36] carried out microarray analysis on human FVMs. Three samples contained active FVMs, and 3 inactive FVMs. They defined activity as the presence of perfused capillaries. The genetic expression of FVMs was compared to RNAs extracted from normal human retinas. Eighty-seven genes were preferentially expressed in FVMs, dominated by ECM genes (COL6A3, THBS2, COL1A1, POSTN, TNC, SERPINE1, VCAN). Active FVMs were enriched for angiogenesis genes (APLN, MMP9, ANGPT2, ESM1) and inactive FVMs for metabolic apoptotic genes (CRYAA highest). The study generated a list of genes that could be targeted therapeutically in the future to slow the progression of FVMs, potentially without affecting normal retinal function. It is worth noting that the FVM samples were surgical specimens collected at two Japanese centres, whereas the reference retinal RNA was obtained from a commercial Caucasian donor pool. Since no ancestry data are available for the FVM patients, population differences cannot be ruled out as a factor influencing the observed variations in genetic expression.

A multimodal characterisation of FVMs using RNA-seq and proteomics carried out by Boneva et al. revealed the presence of 3365 genes upregulated in FVMs versus control samples [37]. The composition was dominated by ECM molecules, endothelial markers and immune regulators. The subsequent immunohistochemical analysis of vitreous hyalocytes revealed expression of alpha-smooth muscle actin, a well-known marker of myofibroblasts. This finding suggested a possible role of hyalocytes in the development of FVMs in PDR.

### 4.4. RNA Methylation

RNA methylation is a post-transcriptional modification method that stabilises RNA and regulates its localisation, transport, splicing and translation [55]. Over 150 types of RNA modifications have been identified to date, but few publications exist on their role in DR [56]. Wang et al. studied 5-methylcytosine (m^5^C) modification in DR vitreous [38]. This particular type of methylation was previously shown to be catalysed by the NSUN family of proteins [57]. Using microarray analysis, the authors found that NSUN2 expression was significantly elevated in the DR vitreous. Further analysis revealed that NSUN2 promoted MUC1 expression by binding to the RNA m^5^C reader ALYREF. This signalling pathway is believed to drive microvascular proliferation, invasion and angiogenesis in DR.

Another study investigated the N6-methyladenosine (m^6^A) demethylation of mRNA in DR, the roles of fat mass and obesity-associated protein (FTO), and the kinesin family member 11 (KIF11) gene [39]. FTO is believed to contribute to vascular endothelial dysfunction in DR. In a previous transcriptomic study, FTO expression was increased in human retinal microvascular endothelial cells (hRECs) cultivated under hyperglycaemic conditions [58]. KIF11 is a tumour biomarker that has also been identified as a potential marker of PDR [59]. However, little is still known about how KIF11 contributes to the progression of DR.

Using meRIP-seq, Lin et al. found that FTO was overexpressed in vitreous PDR, which was followed by the demethylation and overexpression of KIF11. In vitro, this mechanism was shown to enhance proliferation, DNA synthesis, migration, wound healing and tube formation in hRECs. In vivo, it was shown to enhance the development of DR in a streptozotocin-induced rat model. The authors stated that the commercial inhibitor of KIF11 (ispinesib) possesses great potential in the clinical therapy of DR, but the current literature does not support this conclusion yet. It is worth noting that ispinesib’s efficacy as a single agent was not supported by clinical trials of patients with cancers, such as melanoma and head-and-neck squamous cell carcinoma [60,61]. Future studies could investigate ispinesib’s efficacy in the context of PDR to allow for clinical translation.

### 4.5. Non-Coding RNA

Non-coding RNA (ncRNA) refers to a RNA that does not code for a protein but plays a role in gene regulation at epigenetic, transcriptional and post-transcriptional levels [62]. Various types of ncRNAs exist, each having its specific function. The ones mentioned in this review include microRNA (miRNA), long non-coding RNA (lncRNA), tRNA-derived small RNAs (tsRNAs) and circular RNA (circRNA).

Four transcriptomic studies reported on the role of miRNA in PDR vitreous. Yan et al. extracted total RNA from vitreous exosomes. They identified 843 unique miRNAs across all vitreous samples using RNA-seq, with 60 miRNAs showing differential expression between PDR patients and non-diabetic controls [40]. The most significantly downregulated miRNAs in PDR vitreous were miR-204-5p and miR-211-5p, both of which have been shown to play a protective role against diabetic complications. The most upregulated molecules were miR-451a and miR-486-5p, the latter of which is believed to play a role in angiogenesis. However, it is also worth noting that miR-451a is a haemolysis marker [63] and that miR-486-5p is another miRNA expressed by red blood cells [64]. As some of the samples came from eyes with non-clearing vitreous haemorrhage, an important consideration is whether the upregulated molecules were contaminants.

Guo et al. [41] sequenced small RNAs from vitreous, identifying 225 differentially expressed miRNAs (179 upregulated, 46 downregulated). Of these, 10 were chosen for target prediction. Three miRNAs (hsa-miR-24-3p, hsa-miR-197-3p, hsa-miR-3184-3p) were confirmed to be elevated by polymerase chain reaction (PCR) in a second cohort, along with increased vitreous VEGF-A and transforming growth factor-beta (TGF-β) measured by an enzyme-linked immunosorbent assay, with positive Pearson correlations (coefficient range = 0.498–0.586) observed between each miRNA and both growth factors. However, the paper reports only pooled correlations rather than within-group analysis. The reported r values might indicate group separation instead of a relationship within PDR. It would be useful to assess the dose–response relationship within the disease group, meaning PDR patients with higher miR-24-3p levels should exhibit higher VEGF-A levels. This would better support the proposed conclusion that a positive correlation exists between elevated vitreous levels of the three miRNAs and VEGF-A, TGF-β in PDR patients.

Smit-McBride et al. compared miRNA expression across plasma, vitreous and aqueous humour taken from each patient with DR [42]. They profiled 847 miRNA probes on Affymetrix arrays. The results revealed dysregulation of common biomarkers across all aqueous, vitreous and plasma in DR. These included: let-7b, miR-320b, miR-762, miR-4488. The authors noted that their results did not identify the same biomarkers as those reported by previous teams [65,66]. Some miRNAs were from the same family but not the exact same members. Furthermore, the study was underpowered because some subgroups of four to seven subjects fell short of the authors’ own calculated power (*n* = 7). Therefore, further research into miRNA transcriptomics in DR is necessary to identify reliable biomarkers for future clinical use. Moreover, the experimental design section lists the control group as 11 patients with MH or ERM. However, the Supplementary Table S1 from this article also states that the control group included a patient with retinal detachment (the type of detachment was unspecified) and one other patient with an unspecified diagnosis. Interestingly, this is the only study that obtained vitreous samples from patients with NPDR. Supplementary Table S1 lists two of those subjects as having concurrent ERM and vitreomacular traction but the reason for surgery is not stated for the other two patients. This is relevant as patients with NPDR are unlikely to require surgery unless for other reasons (such as ERM peel). Hence, the clinical reason behind sampling two of those patients remains unclear and should be stated as a lack of transparency in the included subjects affects the reproducibility of future studies.

LncRNAs and miRNAs were studied by He et al. using microarray analysis [43]. They found high expression of LINC02099 in PDR vitreous, which was shown to drive hyperglycaemic upregulation of fibronectin, collagen I and collagen IV in Müller cells. Bioinformatic analyses revealed a direct interaction between LINC02099, miR-214-3p and cathepsin S (CTSS). CTSS is believed to drive angiogenesis and inflammation in a hyperglycaemic state [67]. The role of miR-214-3p in DR is still debated. The current literature reports on its antifibrotic properties across diverse disease models [68]. One example is cardiac disease, where miR-214-3p was shown to confer cardioprotective properties against fibrosis and oxidative damage [69,70]. Similarly, it is hypothesised that inhibition of miR-214-3p can contribute to enhanced fibrosis in hyperglycaemic states. Using computational predictions, He et al. found that CTSS is directly suppressed by miR-214-3p. The team concluded that the LINC02099/miR-214-3p/CTSS axis regulates the extracellular matrix (ECM) in DR, contributing to fibrosis.

In another study, Thomas et al. investigated the role of lncRNAs in endothelial–mesenchymal transition (EndMT) in DR [44]. EndMT occurs as a result of hyperglycaemia and involves transdifferentiation of endothelial cells into mesenchymal-like cells [71]. This process leads to the loss of endothelial junctional markers, such as vascular endothelial cadherin and platelet endothelial cell adhesion molecule-1. The resulting increase in vascular permeability causes infiltration of immune cells and leakage of fluid into the affected tissues. The mesenchymal cellular phenotype also drives fibrosis during later stages of the disease [72]. Thomas et al. found that lncRNA H19 prevents EndMT by suppressing TGF-β and its signalling pathways. They found that lncRNA H19 expression was suppressed in DR, driving the EndMT process. However, this paper uses patients with idiopathic MH and unspecified non-diabetic conditions as controls. The lack of a clear statement about which controls were used affects the reproducibility of their results.

Another microarray analysis carried out by Zeng et al. evaluated lncRNA expression in the vitreous of patients with PDR as well as those treated with an anti-VEGF agent [45]. They found 1067 dysregulated non-coding transcripts in PDR vitreous. In particular, five lncRNAs were found to be downregulated: RP11-573J24.1, RP11-787B4.2, RP11-654G14.1, RP11-2A4.3, and RP11-502I4.3. Moreover, the vitreous of patients with PDR after anti-VEGF treatment showed increased expression of RP4-631H13.2 compared to that in untreated patients with PDR. The authors used plasma samples from subjects to compare their transcripts with those of the vitreous. They determined that none of the transcripts verified by PCR showed significant differences in plasma, leading them to conclude that the signal is probably confined to the vitreous. The design is sensible in principle, but it is underpowered to fully support the conclusion made.

In another study involving lncRNAs, Hu et al. [46] isolated exosomes from PDR vitreous using RNA-seq and identified 899 differently expressed lncRNAs, of which LOC100132249 was the most strongly upregulated. Sequencing of matched plasma exosomes revealed no difference, suggesting a retinal origin rather than a systemic one. High-glucose-stimulated human retinal vascular endothelial cells were identified as the likely source. The suggested mechanism involves nuclear LOC100132249 binding to miR-199a-5p and releasing repression of SNAI1, a gene implicated in physiological and pathological ocular neovascularisation [73]. This activates Wnt/β-catenin signalling and results in endothelial dysfunction.

The role of lncRNAs in PDR patients was also investigated by Wang et al. using microarray [47]. They harvested FVMs from patients who underwent pars plana vitrectomy (PPV) with prior intravitreal anti-VEGF injection (conbercept) versus without. Intravitreal injections of anti-VEGF agents prior to PPV were previously described to be beneficial in reducing the incidence of post-operative complications [74]. The study demonstrated that lncRNAs and mRNA expression was affected by conbercept. They found 427 differentially expressed lncRNAs, out of which long intergenic non-coding RNAs (lincRNAs) constituted the largest group. Bioinformatic analysis linked these to metabolic pathways (gluconeogenesis), hypoxia-inducible factor 1 signalling, and inflammatory pathways (including tumour necrosis factor-alpha). It was suggested that understanding the relationship between lncRNAs and anti-VEGF drugs could support the development of future therapies, with lncRNAs serving as potential targets.

One study reported on tsRNAs. Yang et al. performed small RNA sequencing of vitreous samples from patients with PDR and MH [48]. They found 37 differentially expressed tsRNAs and 70 miRNAs. Furthermore, it was established that adenosine monophosphate-activated protein kinase (AMPK) signalling is the leading pathway for tsRNA targets and that Th17 cell differentiation is the leading pathway for miRNA targets. This was the first study that reported tsRNA expression profile from human PDR vitreous.

One study reported on the expression of circRNAs in DR vitreous. CircRNAs can bind to miRNAs and act as their ‘sponges’ to prevent miRNAs from suppressing their target mRNA [75]. Transcriptomic sequencing of PDR vitreous by He et al. demonstrated upregulation of 122 circRNAs and downregulation of 9 [49]. Further bioinformatic analyses predicted that each circRNA could interact with at least 5 miRNAs. Selected PCR testing of 3 randomly chosen miRNAs revealed their downregulation, confirming the suspected effect of circRNA-miRNA crosstalk in PDR vitreous. However, it is worth noting that the control group was varied and comprising one rhegmatogenous retinal detachment (RRD), one MH and one ERM patient. In particular, RRD involves BRB breakdown, making it a suboptimal baseline for a study focused on barrier-associated pathology.

## 5. Discussion

### 5.1. Limitations of Current Vitreous Transcriptomic Research and Future Perspectives

The studies discussed in this review provided new data on the genetic makeup of patients with DR. However, all of the studies were limited by small sample sizes and a lack of healthy controls. The latter is challenging to overcome as vitreous sampling is invasive and carries vision-threatening risks, such as intraocular infection, retinal break, retinal detachment and vitreous haemorrhage [76]. Currently, most researchers utilise patients with idiopathic vitreoretinal pathologies necessitating PPV as controls [33,34,35,77], with some even opting for nonocular tissues [78].

Studies included in this review varied in control subject selection. Seven studies used more than 1 disease in the control group [34,37,39,40,42,44,49]. Three studies did not list the exact type of their control subjects [38,42,44]. Four studies utilised patients with retinal detachment as controls, of which three studies involved RRD [39,40,49] and one did not specify the type of retinal detachment [42]. Heterogeneity in control groups and the lack of specified control subjects likely contribute to the lack of consensus on a single biomarker attributable to PDR across the discussed studies. Moreover, RRD involves BRB breakdown, which makes it a poor choice of control for barrier-associated pathologies (such as DR) [79]. Future studies should aim to utilise the same types of controls in order to allow for meaningful comparison and reproducibility.

Another important limitation of current vitreous transcriptomics research is the lack of a standardised approach to vitreous sampling. Some studies use undiluted vitreous specimens [32], while others propose diluted samples [33]. Parameters such as sample volume or vitrectomy cut rate per minute are not always reported. The latter might be of relevance in preserving the quality of the obtained cells and thus their RNA [80].

There is also the matter of optimising the preservation of RNA material from vitreous tissue following its collection. Samples can be fresh or stored frozen or fixed [81]. Future studies could investigate the correlation between the gene expression profiles of the extracted RNA and the sample preservation method of human vitreous. Bhanja et al. have recently published a detailed protocol on isolating and characterising vitreous immune cell infiltrates for single-cell transcriptomic studies [82], which may improve standardisation of methodology in future projects.

One alternative to obtaining cells from living patients is through the use of organoids, which utilise stem cells to recreate models of various eye structures. There have been reports of organoid models of major eye tissues, including the retina, cornea, lens and conjunctiva [83]. Multiocular organoid models also exist, which allow for recreation of the native-like tissue architecture [84]. Recent studies also integrated organoids into a controlled micro-environment of organ-on-chip (OoC) platforms [85,86]. Microfluidic OoC devices provide a more accurate representation of physiological conditions as they allow for diffusion of nutrients, which mimics vasculature. To the best of our knowledge, there are no published reports on organoid or OoC involving the vitreous humour. Whilst OoC platforms are useful alternatives to human sampling, they are unlikely to succeed in context of in vitro vitreous research. This is because vitreous is virtually acellular and mostly composed of the extracellular matrix [87]. Its transcripts come from exosomes released from the retina, resident hyalocytes and infiltrating inflammatory cells [82,88,89]. Future studies should therefore focus on standardising the existing vitreous sampling protocols and on increasing sample sizes to strengthen the conclusions.

### 5.2. Current Barriers for Clinical Translation

The aim is to identify specific biomarkers responsible for driving PDR that could be used in the future development of targeted therapies. The studies discussed in this article identified numerous biomarkers with little overlap. Therefore, at the present moment, no candidate has been replicated under comparable conditions with blood contamination excluded. Moreover, we are unable to conclude whether the listed biomarkers would also be translatable to patients with NPDR, as samples were only obtained from those with advanced PDR requiring vitrectomy, apart from one study which sampled 4 NPDR patients [42]. Patients with NPDR do not typically require vitrectomy unless for other reasons (such as concurrent MH or ERM). In order to apply the findings to NPDR patients, the future biomarker should ideally be identifiable in the blood of diabetic patients.

Overall, the main barriers to clinical translation of current vitreous transcriptomics research include lack of standardised sampling, lack of standardised controls, and small sample sizes. At the present moment, all of these affect reproducibility, resulting in the inability to identify reliable biomarker candidates.

### 5.3. Limitations of This Review

The abstracts were screened by a single reviewer which lacks independent verification and introduces the risk of potential selection bias. We also trialled using genAI to create initial search strings for PubMed and Embase. We would like to highlight the importance of human checks of the output prior to its execution. Whilst we acknowledge that our prompts were brief (Supplementary File S1), the initial search strings required manual editing to ensure a high-quality search. For instance, the initial PubMed search string contained the abbreviation “PDR”, which was redundant. It was noted that the generated output for the Embase search string contained many more irrelevant terms, resulting in a substantial amount of noise (irrelevant results). For instance, it included the abbreviated terms “DEG or DEGs” in addition to the fully written term “differentially expressed gene*”. When we input the original genAI-generated string into Embase, 111 articles were identified. Following manual editing of the terms to derive the final search string demonstrated in the Methods section, this number was reduced to 79. We analysed the abstracts of the additional 32 articles identified from the genAI output and concluded that none of them met the inclusion criteria. We therefore conclude that using genAI as part of the methodology for review articles is helpful but requires careful human oversight to ensure its efficiency.

## 6. Conclusions

Recent years have seen significant progress in the field of transcriptomics within the vision research community. Furthermore, studies into human vitreous transcriptomics are becoming more prevalent, with promising initial findings. We hope that the integration of transcriptomics into routine clinical practice will become a part of future ophthalmic care. Currently, its implementation is limited by several factors, including a lack of optimised protocols, high cost and a lack of well-powered studies.

## Figures and Tables

**Table 1:** Comparison of microarray and RNA-sequencing methods.

Technology	Principle	Key Considerations	Reference
Microarray	Hybridisation of fluorescently labelled nucleic acid sequences to complementary DNA probes.	Does not allow for novel transcript discoveries. Only detects previously known sequences included in the array. More standardised protocols which improves reproducibility. More cost-effective. Suboptimal detection of genes with low expression. Background noise owing to cross-hybridisation which can generate false positive or false negative results.	[3,28,30]
RNA-seq	High-throughput sequencing-based approach that analyses a complete set of RNA in a given sample. Major types: Bulk RNA-seq: average gene expression across all cells in a given sample; ScRNA-seq: profiles each cell’s unique transcriptome; Spatial RNA-seq: profiles gene expression within the context of its surrounding tissue.	Allows for novel transcript detection, making it more suitable for discovery-based studies. Higher dynamic range which allows identification of both very-high- and very-low-expression genes. Better at detection of splice forms. More expensive than microarray. Numerous protocols which limit reproducibility. Large amount of data generated. Requires expertise in library preparation, sequencing and analysis.	[28]

**Table 2:** Summary of studies on vitreous transcriptomics.

First Author and Publication Year	Transcriptomic Method	Vitreous Sample (*n*) for Transcriptomics Analysis	Control Group (*n*) for Transcriptomics Analysis	Key Findings	Database	Reference
Li et al. (2025)	RNA-seq	PDR vitreous (*n* = 5)	MH vitreous (*n* = 5)	ADAMTS13 concentrations were higher in PDR vitreous compared to MH vitreous. ADAMTS13 knockdown reduced retinal vascular leakage, cell migration, and neovascularisation in vitro and vivo. ADAMTS13 drives angiogenesis via the PLK-1/Ang-2 axis, independent of VEGF.	PubMed; Embase	[32]
Haliyur et al. (2024)	scRNA-seq	PDR vitreous (*n* = 6)	Peripheral venous blood samples from the same PDR patients (*n* = 5)	PDR vitreous was primarily composed of T cells, with activity and proportions distinct from T cells within the peripheral blood. Neutrophils were absent in PDR vitreous.	PubMed; Embase	[33]
Boneva et al. (2024)	RNA-seq	PDR vitreous (*n* = 8)	ERM vitreous (*n* = 4) MH vitreous (*n* = 5)	PDR hyalocytes showed enhanced inflammatory response (IL-6), a proangiogenic shift (increased angiopoietin-2), and upregulated haemoglobin-catabolism genes. Porcine hyalocytes phagocytosed red blood cells, indicating a role of hyalocytes in clearing vitreous haemorrhage.	PubMed; Embase	[34]
Hu et al. (2022)	scRNA-seq	PDR FVMs (*n* = 5)	scRNA-seq GEO dataset of retinal microglia and peripheral blood mononuclear cells	Profibrotic microglia were identified as the main cell population in PDR FVMs.	PubMed; Embase	[35]
Ishikawa et al. (2015)	Microarray	PDR FVMs (*n* = 6)	Human retinas (*n* = 3)	87 genes were preferentially expressed in FVMs, dominated by ECM genes (COL6A3, THBS2, COL1A1, POSTN, TNC, SERPINE1, VCAN). Active FVMs were enriched for angiogenesis genes (APLN, MMP9, ANGPT2, ESM1) and inactive FVMs for metabolic apoptotic genes (CRYAA highest).	PubMed; Embase	[36]
Boneva et al. (2021)	RNA-seq	PDR FVMs (*n* = 7)	ERM vitreous (*n* = 10) MH vitreous (*n* = 7)	Transcriptional and proteomic analysis of PDR FVMs revealed presence of endothelial cells, macrophages, myofibroblasts and ECM proteins. Vitreous hyalocytes present in PDR samples was found to express alpha-smooth muscle actin, indicative of the role of hyalocytes in the development of FVMs.	PubMed; Embase	[37]
Wang et al. (2024)	Microarray	DR vitreous (*n* = 20)	Vitreous sample from unspecified non-diabetic condition (*n* = 20)	NSUN2 and RNA m^5^C methylation were elevated in DR. NSUN2 promoted MUC1 expression by binding the m^5^C reader ALYREF. NSUN2 or ALYREF knockdown reduced DR lesions.	PubMed; Embase	[38]
Lin et al. (2026)	MeRIP-seq	PDR vitreous (*n* = 90)	RRD vitreous (*n* = 26) MH vitreous (*n* = 30) ERM vitreous (*n* = 34)	FTO was overexpressed in vitreous PDR which was followed by the demethylation and overexpression of KIF11. This mechanism was linked to progression of DR in a diabetic mouse model.	PubMed	[39]
Yan et al. (2025)	RNA-seq	PDR vitreous (*n* = 23)	RRD vitreous (*n* = 14) MH vitreous (*n* = 2) ERM vitreous (*n* = 1)	Sequencing identified 843 miRNAs, with 60 differentially expressed in PDR vitreous (28 upregulated, 32 downregulated). Most upregulated: miR-451a, miR-486-5p; most downregulated: miR-204-5p, miR-211-5p.	PubMed	[40]
Guo et al. (2021)	RNA-seq	PDR vitreous (*n* = 5)	MH vitreous (*n* = 5)	Three miRNAs (hsa-miR-24-3p, hsa-miR-197-3p and hsa-miR-3184-3p) were found to be elevated in PDR vitreous. They were positively correlated with elevated levels of VEGF and TGF-β in PDR.	Embase	[41]
Smit-McBride et al. (2020)	Microarray	T1DM PDR vitreous (*n* = 5) T2DM PDR vitreous (*n* = 7) T2DM NPDR vitreous (*n* = 4)	MH vitreous (*n* = 1) Retinal detachment vitreous (*n* = 1) ERM vitreous (*n* = 8) Vitreous sample from unspecified non-diabetic condition (*n* = 1); Samples of aqueous, vitreous and plasma were collected from most patients enrolled in the study.	Most dysregulated miRNAs were upregulated in aqueous and vitreous of DM patients but downregulated in plasma. Common biomarkers dysregulated in both aqueous and vitreous in DR included: let-7b, miR-320b, miR-762, miR-4488.	PubMed	[42]
He et al. (2025)	Microarray	PDR vitreous (*n* = 3)	MH vitreous (*n* = 3)	LINC02099 was the most highly expressed lncRNA in PDR vitreous. It sponges miRNA-214-3p, which targets Cathepsin S (CTSS). The LINC02099/miRNA-214-3p/CTSS axis drives ECM deposition, contributing to retinal fibrosis in DR.	PubMed	[43]
Thomas et al. (2019)	RNA-seq	PDR vitreous (*n* = 8)	Vitreous from MH and other unspecified non-diabetic conditions (*n* = 5)	Expression of H19 was downregulated under high-glucose conditions. H19 overexpression prevented glucose-induced endothelial–mesenchymal transition. PDR vitreous showed H19 downregulation.	PubMed	[44]
Zeng et al. (2023)	Microarray	PDR vitreous (*n* = 3) PDR vitreous pretreated with anti-VEGF intravitreal injection 3–7 days before surgery (*n* = 3)	MH vitreous (*n* = 3)	1067 differentially expressed non-coding RNA transcripts were identified in vitreous of PDR patients and not in those with MH. Five long non-coding RNAs were downregulated: RP11-573J24.1, RP11-787B4.2, RP11-654G14.1, RP11-2A4.3, RP11-502I4.3. In addition, RP4-631H13.2 was significantly upregulated in the PDR vitreous treated with anti-VEGF.	PubMed; Embase	[45]
Hu et al. (2023)	RNA-seq	PDR vitreous (*n* = 25)	MH vitreous (*n* = 40)	The most upregulated exosomal transcript was lncRNA LOC100132249. It acted as a sponge for miR-199a-5p, relieving repression of SNAI1 and activating Wnt/β-catenin signalling. This process promotes endothelial-mesenchymal transition and angiogenesis both in vitro and in an oxygen-induced retinopathy mouse model.	Background reading	[46]
Wang et al. (2019)	Microarray	PDR FVMs pretreated with anti-VEGF intravitreal injection 7 days before surgery (*n* = 10)	PDR FVMs (*n* = 10)	Differential expression was found in 427 lncRNAs, out of which lincRNAs constituted the largest group. Intravitreal conbercept was given before PPV altered the expression of lncRNAs and mRNAs in the FVMs, with the affected transcripts linked to HIF-1 signalling, as well as inflammatory and metabolic pathways.	PubMed; Embase	[47]
Yang et al. (2022)	RNA-seq	PDR vitreous (*n* = 4)	MH vitreous (*n* = 4)	Differential expression was noted in 37 tRNA-derived RNAs and 70 miRNAs, with selected hits validated by RT-qPCR in the same samples. Predicted target genes were enriched for AMPK signalling (tsRNAs) and Th17 cell differentiation (miRNAs).	Background reading	[48]
He et al. (2020)	RNA-seq	PDR vitreous (*n* = 3)	RRD vitreous (*n* = 1) MH vitreous (*n* = 1) ERM vitreous (*n* = 1)	Compared to control group, PDR vitreous had 122 upregulated and 9 downregulated circRNAs, as well as 818 upregulated and 864 downregulated mRNAs. Bioinformatic analysis showed that each circRNA could absorb at least five miRNAs.	PubMed; Embase	[49]

Abbreviations: AMPK, AMP-activated protein kinase; Ang-2, angiopoietin-2; circRNA, circular RNA; DM, diabetes mellitus; DR, diabetic retinopathy; ECM, extracellular matrix; ERM, epiretinal membrane; FVM, fibrovascular membrane; GEO, Gene Expression Omnibus; HIF-1, hypoxia-inducible factor 1; IL-6, interleukin-6; KIF11, kinesin family member 11; lincRNA, long intergenic non-coding RNA; lncRNA, long non-coding RNA; m^5^C, 5-methylcytosine; MeRIP-seq, methylated RNA immunoprecipitation sequencing; MH, macular hole; miRNA/miR, microRNA; mRNA, messenger RNA; NPDR, non-proliferative diabetic retinopathy; PDR, proliferative diabetic retinopathy; PLK-1, polo-like kinase 1; PPV, pars plana vitrectomy; RNA-seq, RNA sequencing; RRD, rhegmatogenous retinal detachment; RT-qPCR, reverse transcription quantitative polymerase chain reaction; scRNA-seq, single-cell RNA sequencing; T1DM, type 1 diabetes mellitus; T2DM, type 2 diabetes mellitus; TGF-β, transforming growth factor beta; Th17, T helper 17; tRNA, transfer RNA; tsRNA, tRNA-derived small RNA; VEGF, vascular endothelial growth factor.

## Data Availability

No new data were created or analysed in this study. Data sharing is not applicable to this article.

## Supplementary Materials

The following supporting information can be downloaded at: https://www.mdpi.com/article/10.3390/biology15211997/s1, File S1: supplementary methods.

## Abbreviations

The following abbreviations are used in this manuscript:

ADAMTS13	A disintegrin-like and metalloprotease with thrombospondin type 1 motif, 13
AGE	Advanced glycation end products
AMPK	Adenosine monophosphate-activated protein kinase
circRNA	Circular RNA
DM	Diabetes mellitus
DR	Diabetic retinopathy
EndMT	Endothelial–mesenchymal transition
ERM	Epiretinal membrane
FVM	Fibrovascular membrane
genAI	Generative artificial intelligence
iBRB	Inner blood–retinal barrier
lncRNA	Long non-coding RNA
m^5^C	5-methylcytosine
MH	Macular hole
MIP-1α	Macrophage inflammatory protein-1α
miRNA	MicroRNA
ncRNA	Non-coding RNA
NPDR	Non-proliferative diabetic retinopathy
OoC	Organ-on-chip
PCR	Polymerase chain reaction
PPV	Pars plana vitrectomy
PDR	Proliferative diabetic retinopathy
RNA-seq	RNA sequencing
RRD	Rhegmatogenous retinal detachment
scRNA-seq	Single-cell RNA sequencing
TGF-β	Transforming growth factor-beta
tsRNA	tRNA-derived small RNAs
VEGF	Vascular endothelial growth factor
