# Supplementary material for: Vitreous Transcriptomics in Diabetic Retinopathy: Current Research and Future Prospects
Source: Biology (Basel). 2026 Nov 4;15(21):1997. doi: 10.3390/biology15211997 (PMC13762205; doi:10.3390/biology15211997)
Supplement: Supplementary file 1 [file biology-15-01997-s001.zip › biology-4593655-supplementary.pdf]

File S1. Supplementary methods

Generative artificial intelligence (genAI) chat (Claude Opus 5, Anthropic, accessed 27th August 2026) was utilised to create the initial search strings for the literature search in PubMed and Embase (Ovid) platforms.

To create the search string for PubMed, the following prompt was used:

"I want to write a literature review on vitreous transcriptomics in diabetic retinopathy. Come up with search terms for PubMed that I can use to carry out my literature search."

The resulting output was as below:

("Diabetic Retinopathy"[MeSH] OR diabetic retinopathy[tiab] OR proliferative diabetic retinopathy[tiab] OR PDR[tiab]) AND ("Vitreous Body"[MeSH] OR vitreous[tiab] OR vitreous humor[tiab] OR vitreous fluid[tiab]) AND ("Transcriptome"[MeSH] OR "Gene Expression Profiling"[MeSH] OR "Sequence Analysis, RNA"[MeSH] OR transcriptomic*[tiab] OR transcriptome[tiab] OR "RNA-seq"[tiab] OR "RNA sequencing"[tiab] OR "gene expression profiling"[tiab] OR microarray[tiab] OR "differentially expressed genes"[tiab])

Subsequently, another prompt was run to create a search string for Embase (Ovid).

"I am writing a literature review article on vitreous transcriptomics in diabetic retinopathy. I already carried out PubMed search using the following search string: ("Diabetic Retinopathy"[MeSH] OR diabetic retinopathy[tiab] OR proliferative diabetic retinopathy[tiab] OR PDR[tiab]) AND ("Vitreous Body"[MeSH] OR vitreous[tiab] OR vitreous humor[tiab] OR vitreous fluid[tiab]) AND ("Transcriptome"[MeSH] OR "Gene Expression Profiling"[MeSH] OR "Sequence Analysis, RNA"[MeSH] OR transcriptomic*[tiab] OR transcriptome[tiab] OR "RNA-seq"[tiab] OR "RNA sequencing"[tiab] OR "gene expression profiling"[tiab] OR microarray[tiab] OR "differentially expressed genes"[tiab]). I need to carry out search on Ovid embase. I would like you to create a good search string that will identify articles across three concept blocks: diabetic retinopathy, vitreous, and transcriptomic methodology."

The following search string was generated:

1. exp diabetic retinopathy/

2. (diabetic retinopath* or "proliferative diabetic retinopath*" or PDR).ti,ab,kw.

3. 1 or 2

4. exp vitreous body/

5. (vitreous or "vitreous humor" or "vitreous humour" or "vitreous fluid" or "vitreous bod*").ti,ab,kw.

6. 4 or 5

7. transcriptomics/ or transcriptome/ or gene expression profiling/ or RNA sequencing/ or RNA sequence/ or microarray analysis/ or DNA microarray/

8. (transcriptomic* or transcriptome* or "RNA seq*" or RNAseq or "RNA sequencing" or "gene expression profil*" or microarray* or "differentially expressed gene*" or DEG or DEGs).ti,ab,kw.

9. 7 or 8

10. 3 and 6 and 9

Following the generated outputs, both search strings were manually revised and amended to better suit the scientific search strategy. The final edited outputs are included in the methods section of the article.
